# Non-allergic severe asthma: is it really always non-allergic? The IDENTIFY project

**DOI:** 10.1186/s13223-020-00489-z

**Published:** 2020-11-02

**Authors:** Dirk Koschel, Claudia Mailänder, Inessa Schwab Sauerbeck, Jens Schreiber

**Affiliations:** 1 Department of Internal Medicine and Pneumology, Fachkrankenhaus Coswig, Lung Center, Coswig, Germany; 2 Divison of Pneumology, Medical Department I, University Hospital Carl Gustav Carus, Technische Universität Dresden, Dresden, Germany; 3 Clinical Research, Respiratory, Novartis Pharma GmbH, Nürnberg, Germany; 4 Dept of Pneumonology, University Hospital, Otto-Von-Guericke-University Magdeburg, Leipziger Strasse 44, 39120 Magdeburg, Germany

**Keywords:** Immunoglobulin E, Allergens, Perennial, Sensitization

## Abstract

**Background:**

This differential diagnosis of allergic vs non-allergic asthma is typically made on the basis of sensitization to allergens, such that absence of sensitization could result in a patient being managed as having non-allergic asthma. In Germany, the number of specific allergen tests is limited and non-standardized (across clinicians and laboratories), with the potential for false negative diagnoses. IDENTIFY aimed to gain data on sensitizations toward aeroallergens in patients with severe asthma who had tested negative to perennial aeroallergens in previous tests.

**Methods:**

This was a single visit, non-randomized, non-interventional study conducted in 87 centers across Germany. The only inclusion criteria were that patients had to be adults (at least 18 years of age) with a diagnosis of severe asthma (receiving at least Global Initiative for Asthma Step IV therapy), and who had previously tested negative to perennial aeroallergens. Patients were then tested for sensitization to a panel of 35 perennial aeroallergens, with positive sensitization indicated by CAP ≥ 0.35 kU/L.

**Results:**

Of 588 patients recruited, 454 had complete and valid data, and had previously tested negative to perennial aeroallergens. Overall, 43.6% of the analyzed patients tested positive for at least one of the included aeroallergens, with 18.7% testing positive for three or more, and 4.2% positive for more than ten. The five most common sensitizations were to *Staphylococcus aureus* enterotoxin B, *Aspergillus fumigatus*, *Candida albicans*, *Dermatophagoides farinae*, and *Rhizopus nigricans*, each of which tested positive in at least 9.7% of the population.

**Conclusions:**

In this group of patients being managed as having non-allergic asthma (and who had all previously tested negative to perennial aeroallergens), a high proportion tested positive to a broad panel of aeroallergens. A diagnosis of allergic asthma therefore cannot be excluded purely on the basis of standard aeroallergen panels.

## Background

With the increasing numbers of biologic therapies available for the management of severe asthma, accurate differential diagnosis of allergic and non-allergic asthma is becoming increasingly important. This differential diagnosis is typically made on the basis of the presence or absence of sensitization to allergens, with eligibility for an anti-immunoglobulin E (IgE) therapy based in part on sensitization to perennial aeroallergens [1]. As a consequence, absence of sensitization could result in a patient being managed as having non-allergic asthma.

Testing for sensitization to aeroallergens typically involves either skin prick testing or a specific IgE antibody test. In Germany, skin prick testing is only performed by allergen specialists, resulting in limited access. Although specific IgE antibody tests are more widely available, they are limited to eight allergens every 3 months by statutory health insurers (public payers, who provide health insurance to approximately 90% of the German population), and so in clinical practice a diagnosis of allergic asthma is often limited to this number of allergens. There is frequently no clear rationale to the selection of these eight aeroallergens, and importantly the panel is not standardized across physicians or laboratories. This limited and non-standardized testing therefore has the potential for false negative diagnoses—in other words indicating the absence of sensitization.

The aim of the current study (IDENTIFY) was to gain data on sensitizations toward aeroallergens in patients with severe asthma who had tested negative to perennial aeroallergens in previous tests—i.e., patients being managed in clinical practice as not being indicated anti-IgE therapy for allergic asthma. The study therefore sought to determine whether the use of a broader spectrum of aeroallergens could improve the diagnostic yield. In turn, this would be relevant to patients as identification of a specific allergen would help inform such approaches as avoidance strategies. To our knowledge this is the first study to conduct such analyses, although data from an initial subset of 362 patients has been previously published [2]. This initial publication focused on the subset of patients who were sensitized to *Staphylococcus aureus* enterotoxin A (approximately 15%) and B (approximately 25%). The current analyses expand on this initial publication [2].

## Materials and methods

This was a single visit, non-randomized, non-interventional study. The primary objective was to identify previously undetected sensitization towards perennial aeroallergens in patients with severe asthma, using an extended (35 aeroallergen) radioallergosorbent test (RAST) or ImmunoCAP test. All investigators were the patients’ own treating physicians, who therefore had full access to their medical records. Patients who met the inclusion criteria had their demographic and disease characteristics documented (either by direct assessment or from medical records), and a sample of blood drawn. This sample was then tested for sensitization to a panel of 35 perennial aeroallergens (Additional file 1: Table S1). The potential positive sensitization to the aeroallergens was tested using Immulite® (Siemens, Erlangen, Germany) or ImmunoCAP® (ThermoFisher Scientific, Freiburg, Germany), with positive sensitization indicated by CAP ≥ 0.35 kU/L.

The only inclusion criteria applied were that patients had to be adults (at least 18 years of age) with a diagnosis of severe asthma (receiving at least Global Initiative for Asthma [GINA] Step IV therapy), and who had previously tested negative to perennial aeroallergens in skin prick tests, RAST, or both. All patients had to provide written informed consent, and the protocol was approved by the Ethics Committee of the Faculty of Medicine of the University of Magdeburg prior to project start and by ethics committees of the participating sites. All patients provided written informed consent prior to any study-related procedure. The investigators were all board-certified pneumonologists or allergologists.

The study was not formally powered. It was estimated that 600 patients would be sufficient for the study aim. Data were analyzed overall and in the following subgroups: obese patients (defined by body mass index > 30 kg/m^2^); exacerbations in the 12 months prior to enrolment (≥ or < 2); *Staphylococcus aureus* enterotoxin A or B sensitization (negative to both, and positive to either); positive for at least one aeroallergen; and maintenance oral corticosteroid use (no or yes). All results are presented descriptively.

## Results

The study was conducted between February 2014 and October 2018 in 87 centers distributed across Germany (> 95% of which were office-based physicians, with the remainder being outpatient asthma clinics). A total of 588 patients were recruited, with complete and valid data from 520. Of these, 57 had previously not had a RAST or skin prick test, and nine had previously tested positive to at least one aeroallergen. Both of these groups were excluded from the analyses to leave 454 patients (313 of whom had previously had a RAST and 346 a skin prick test, such that 205 patients had previously had both a RAST and a skin prick test). The majority of patients were female, with mean age of 56.4 years and mean asthma duration 13.2 years, with 92.3% diagnosed at the age of 12 years or older (Additional file 1: Table S2). Only 12.1% met the GINA definition of controlled asthma at the time of our analyses, with approximately three quarters having had at least one exacerbation in the previous year and almost one third receiving maintenance systemic steroids.

### Proportion of patients with positive specific IgE results

**Fig. 1:**
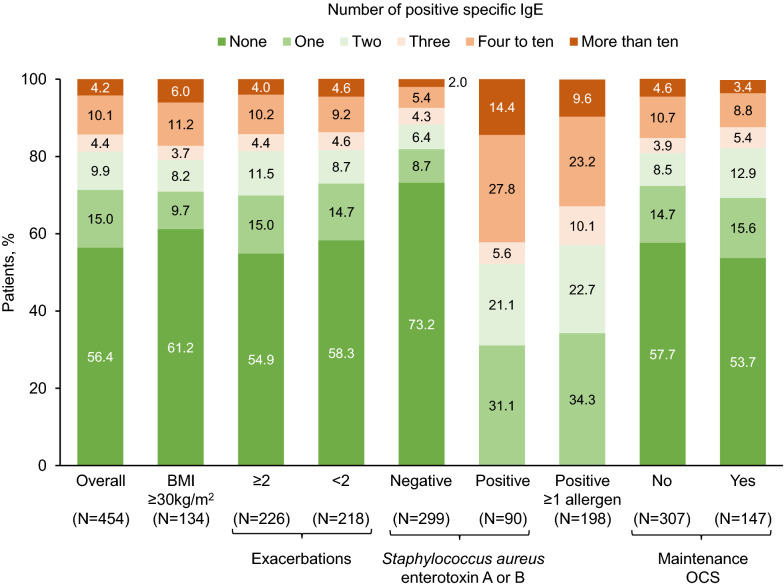
Number of patients with positive specific IgE results. *IgE* immunoglobulin E, *BMI* body mass index, *OCS* oral corticosteroid

Overall, 56.4% of the analyzed patients tested negative for all of the included aeroallergens (Fig. 1). However, 18.7% of the patients tested positive for three or more of the aeroallergens, with 4.2% positive for more than ten. Results were similar within the eight subgroups, with between 2.0 and 14.4% testing positive for more than ten aeroallergens—the highest proportion being in the subgroup of patients testing positive for sensitization to *Staphylococcus aureus* enterotoxin A or B.

### Most common perennial aeroallergens

In the overall population, the five most common sensitizations were to *Staphylococcus aureus* enterotoxin B, *Aspergillus fumigatus*, *Candida albicans*, *Dermatophagoides farinae*, and *Rhizopus nigricans*, each of which tested positive in at least 9.7% of the population (Table 1). With the exception of the subgroup testing positive for *Staphylococcus aureus* enterotoxin A or B, these five most common sensitizations included the top three in all of the subgroups. Thirty-three patients tested positive for *Staphylococcus aureus* enterotoxin A or B but no other aeroallergen (eight for A, 20 for B, five for both).

**Table 1:** Most common aeroallergens (top 10 by incidence), overall and in subgroups

Specific IgE	Overall (N = 454)		BMI ≥ 30 kg/m^2^ (N = 134)		Exacerbations				*Staphylococcus aureus* enterotoxin A or B				Positive for at least one aeroallergen (N = 198)		Maintenance OCS			
					≥ 2 (N = 226)		< 2 (N = 218)		Negative (N = 299)		Positive (N = 90)				No (N = 307)		Yes (N = 147)	
	#	n (%)	#	n (%)	#	n (%)	#	n (%)	#	n (%)	#	n (%)	#	n (%)	#	n (%)	#	n (%)
*Staphylococcus aureus* enterotoxin B	1	72 (15.9)	1	17 (12.7)	1	41 (18.1)	1	30 (13.8)	–	–	1	72 (80.0)	1	72 (36.4)	1	45 (14.7)	1	27 (18.4)
*Aspergillus fumigatus*	2	51 (11.2)	9	11 (8.2)	2	30 (13.3)	6	20 (9.2)	1	26 (8.7)	6	18 (20.0)	2	51 (25.8)	3 =	31 (10.1)	2	20 (13.6)
*Candida albicans*	3	48 (10.6)	2	16 (11.9)	3 =	23 (10.2)	2	24 (11.0)	3	20 (6.7)	3 =	24 (26.7)	3	48 (24.2)	3 =	31 (10.1)	4	17 (11.6)
*Dermatophagoides farinae*	4 =	44 (9.7)	3 =	14 (10.4)	5 =	21 (9.3)	3	22 (10.1)	2	23 (7.7)	7	16 (17.8)	4 =	44 (22.2)	2	36 (11.7)	9	8 (5.4)
*Rhizopus nigricans*	4 =	44 (9.7)	8	12 (9.0)	5 =	21 (9.3)	4 =	21 (9.6)	NA	8 (2.7)	3 =	24 (26.7)	4 =	44 (22.2)	7 =	26 (8.5)	3	18 (12.2)
*Staphylococcus aureus* enterotoxin A	6	41 (9.0)	6 =	13 (9.7)	3 =	23 (10.2)	7 =	18 (8.3)	–	–	2	41 (45.6)	6	41 (20.7)	6	27 (8.8)	5	14 (9.5)
Moth	7	38 (8.4)	3 =	14 (10.4)	7	16 (7.1)	4 =	21 (9.6)	6 =	16 (6.4)	5	19 (21.1)	7	38 (19.2)	5	29 (9.4)	7 =	9 (6.1)
*Dermatophagoides pteronyssinus*	8	31 (6.8)	10 =	10 (7.5)	9 =	13 (5.8)	7 =	18 (8.3)	6 =	16 (6.4)	9	12 (13.3)	8	31 (15.7)	7 =	26 (8.5)	NA	5 (3.4)
Cockroach	9 =	30 (6.6)	3 =	14 (10.4)	8	14 (6.2)	NA	15 (6.9)	9 =	14 (4.7)	8	15 (16.7)	9 =	30 (15.2)	NA	20 (6.5)	6	10 (6.8)
*Dermatophagoides microceras*	9 =	30 (6.6)	6 =	13 (9.7)	9 =	13 (5.8)	9 =	16 (7.3)	6 =	16 (6.4)	10	11 (12.2)	9 =	30 (15.2)	9	25 (8.1)	NA	5 (3.4)
Cat dander	NA	29 (6.4)	NA	7 (5.2)	9 =	13 (5.8)	NA	15 (6.9)	5	18 (6.0)	NA	6 (6.7)	NA	29 (14.6)	NA	20 (6.5)	7 =	9 (6.1)
Dog dander	NA	29 (6.4)	NA	5 (3.7)	NA	12 (5.3)	9 =	16 (7.3)	4	19 (6.4)	NA	5 (5.6)	NA	29 (14.6)	10	23 (7.5)	NA	6 (4.1)
Tyrophagus putrescentiae	NA	25 (5.5)	10 =	10 (7.5)	NA	11 (4.9)	NA	13 (6.0)	9 =	14 (4.7)	NA	10 (11.1)	NA	25 (12.6)	NA	18 (5.9)	10	7 (4.8)
Acarius siro	NA	22 (4.8)	10 =	10 (7.5)	NA	10 (4.4)	NA	12 (5.5)	NA	11 (3.7)	NA	10 (11.1)	NA	22 (11.1)	NA	17 (5.5)	NA	5 (3.4)
Penicillium chrysogenum	NA	18 (4.0)	10 =	10 (7.5)	NA	9 (4.0)	NA	9 (4.1)	NA	9 (3.0)	NA	9 (10.0)	NA	18 (9.1)	NA	14 (4.6)	NA	4 (2.7)

Data are for the number (%) of patients with CAP > 0. # is position in top 10 for each aeroallergen, with NA indicating that the aeroallergen is not in the top 10 for that group. Rows are sorted by percentage of patients in the overall population. *IgE* immunoglobulin E, *BMI* body mass index, *OCS* oral corticosteroid

## Discussion

All of the analyzed population had previously tested negative to perennial aeroallergens in RAST, skin prick tests, or both, and so were being managed in clinical practice as not being indicated anti-IgE therapy for allergic asthma. Despite this, just under half of the overall population showed an IgE-mediated sensitization to at least one perennial aeroallergen, with nearly 5% testing positive for more than ten. This pattern was similar in most of the subgroups—with the obvious exceptions of the subgroups testing positive for at least one aeroallergen, or positive for *Staphylococcus aureus*, in which more than a third of patients tested positive for four or more aeroallergens. For some of these patients, the results therefore provide an opportunity to manage their asthma by avoiding exposure to previously unrecognized allergens. Importantly, all of the recruited patients were receiving care from specialized pneumonologists or allergologists (in Germany a specialism of ‘allergology’ is not recognized, and so all such physicians will have a different primary specialism, such as dermatology, with allergology as a secondary interest).

Although there was good consistency across subgroups for the most common aeroallergens, patients tested positive for a broad range, suggesting that a broad panel (potentially all 35 included in the current study) would be required to exclude the presence of allergic asthma. The results from the current analyses are consistent with data pooled from three studies, one of which was IDENTIFY, in which there was a similarly high proportion of patients who did not test positive to the standard allergen panel but who tested positive for a broader panel [3]. Given the patients recruited into IDENTIFY had previously tested negative to perennial aeroallergens in standard testing (and were therefore presumably being treated as having non-allergic asthma), these results emphasize the importance of identification of potential causative allergen(s) prior to deciding which allergens to include in a sensitization test, rather than just employing a laboratory’s standard panel—with any panel complemented by individual allergens based on a patient’s history and exposure. Indeed, the data have led to a broadening in the standard testing panel used in Germany.

This was a population that had been predominantly diagnosed at the age of at least 12 years. There is a perception that allergic asthma is more common in childhood-diagnosed asthma, whereas in those diagnosed in adulthood non-allergic asthma is more common [4]. The high prevalence of sensitization to aeroallergens in these analyses suggest that a differential diagnosis cannot be made only on the basis of age of onset, and that a high percentage of adults with asthma could be allergic.

To our knowledge, this is the first study to evaluate the prevalence of sensitivity to perennial aeroallergens in patients with severe asthma who have previously tested negative. Our analyses do have some limitations. Although study sites were distributed across Germany, these data cannot necessarily be extrapolated to other countries—similar analyses would be required to identify the most common aeroallergens (although it is likely that a similarly broad panel of aeroallergens would be needed). In addition, we tested sensitization only, and a positive response does not necessarily mean that there would be a clinically relevant response to that allergen in each of these individuals. Indeed, prior to initiation of any anti-IgE therapy, such individuals would need to undergo a series of other tests that were not included in this study. The clinical relevance of the findings therefore need to be evaluated in a future, appropriately designed study.

## Conclusions

In this group of patients being managed as having non-allergic asthma (and who had all previously tested negative to perennial aeroallergens), we identified a high proportion who tested positive to a broad panel of aeroallergens. A diagnosis of allergic asthma therefore cannot be excluded purely on the basis of standard aeroallergen panels, and in patients who do not react to such a standard panel, testing for a broader range of aeroallergens (informed by a patient’s history and exposure) should be considered before allergic asthma is excluded.

## Supplementary information

**Additional file 1: Table S1.** Perennial aeroallergens used for sensitization testing. **Table S2.** Demography and disease characteristics.

## Data Availability

Data are available following submission of a valid research proposal to the corresponding author.

## Abbreviations

IgE: Immunoglobulin E
RAST: Radioallergosorbent test
GINA: Global Initiative for Asthma
BMI: Body mass index
OCS: Oral corticosteroid
