# Supplementary material for: Non-allergic severe asthma: is it really always non-allergic? The IDENTIFY project
Source: Allergy Asthma Clin Immunol. 2020 Nov 2;16:92. doi: 10.1186/s13223-020-00489-z (PMC7607813; doi:10.1186/s13223-020-00489-z)
Supplement: Supplementary file 1 — Additional file 1: Table S1. Perennial aeroallergens used for sensitization testing. Table S2. Demography and disease characteristics. [file 13223_2020_489_MOESM1_ESM.docx]

# Non-allergic severe asthma – is it really always non-allergic? The IDENTIFY project

Dirk Koschel, Claudia Mailänder, Inessa Schwab Sauerbeck, Jens Schreiber

## Additional file 1

## Methods

Supplementary Table 1. Perennial aeroallergens used for sensitization testing.

| **Class of aeroallergen** | **Species of aeroallergen** | **Name of aeroallergen** |
| --- | --- | --- |
| House dust mites | *Dermatophagoides pteronyssinus* | d1 |
|  | *Dermatophagoides farinae* | d2 |
|  | *Dermatophagoides microceras* | d3 |
| Storage mites | *Lepidoglyphus destructor* | d71 |
|  | *Glycophagus domesticus* | d73 |
|  | *Tyrophagus putrescentiae* | d72 |
|  | *Acarus siro* | d70 |
|  | *Euroglyphus maynei* | d74 |
|  | *Blomia tropicalis* | d201 |
| Molds/microorganisms | *Aspergillus fumigatus* | m3 |
|  | *Aspergillus niger* | m207 |
|  | *Staphylococcus aureus* Enterotoxin A | m80 |
|  | *Staphylococcus aureus* Enterotoxin B | m81 |
|  | *Cladosporium herbarum* | m2 |
|  | *Penicillium chrysogenum* | m1 |
|  | *Alternaria alternata* | m6 |
|  | *Aspergillus flavus* | m228 |
|  | *Aspergillus terreus* | m36 |
|  | *Mucor racemosus* | m4 |
|  | *Rhizopus nigricans* | m11 |
|  | *Phoma betae* | m13 |
|  | *Aureobasidium pullulans* | m12 |
|  | *Penicillium glabrum* | m209 |
|  | *Candida albicans* | m5 |
|  | *Setomelanomma rostrata* | m8 |
|  | *Chaetomium globosum* | m208 |
| Animal epithelia | Cat dander | e1 |
|  | Dog dander | e5 |
|  | Horse dander | e3 |
|  | Bovine epithelia | e4 |
|  | Guinea pig epithelia | e6 |
|  | Hamster epithelia | e84 |
|  | Mice epithelia | e71 |
| Insects | Moth (*Bombyx mori*) | i8 |
|  | Cockroach | i6 |

## Results

Supplementary Table 2. Demography and disease characteristics.

|  | **All patients (N=454)** |
| --- | --- |
| Gender, male, n (%) | 170 (37.4) |
| Age, years, mean±SD | 56.4±13.7 |
| Body mass index, kg/m^2^, mean±SD | 27.6±5.9 |
| Smoking status, n (%) |  |
| Non-smoker | 238 (52.4) |
| Ex-smoker | 178 (39.2) |
| Current smoker | 38 (8.4) |
| Asthma duration, years, mean±SD | 13.2±11.4 |
| Age at diagnosis, n (%) |  |
| <12 years | 35 (7.7) |
| ≥12 years | 417 (92.3) |
| GINA asthma control level, n (%) |  |
| Controlled | 55 (12.1) |
| Partly controlled | 168 (37.0) |
| Uncontrolled | 231 (50.9) |
| Exacerbations in the previous 12 months, n (%) |  |
| 0 | 112 (24.7) |
| 1 | 106 (23.3) |
| ≥2 | 226 (49.8) |
| Total IgE, kU/L, mean±SD | 422.0±2634.3 (n=372) |
| Asthma maintenance therapy, n (%) |  |
| Inhaled corticosteroid | 448 (98.7) |
| Long-acting beta2 agonist | 436 (96.0) |
| Long-acting muscarinic antagonist | 243 (53.5) |
| Systemic steroid | 147 (32.4) |
| Leukotriene receptor antagonist | 150 (33.0) |
| Xanthine derivative | 36 (7.9) |

Abbreviations: GINA, Global Initiative for Asthma; IgE, immunoglobulin E
